# A Multi-Objective Partition Method for Marine Sensor Networks Based on Degree of Event Correlation

**DOI:** 10.3390/s17102168

**Published:** 2017-09-21

**Authors:** Dongmei Huang, Chenyixuan Xu, Danfeng Zhao, Wei Song, Qi He

**Affiliations:** College of Information Technology, Shanghai Ocean University, Shanghai 201306, China; dmhuang@shou.edu.cn (D.H.); cyx_xu@163.com (C.X.); wsong@shou.edu.cn (W.S.); qihe@shou.edu.cn (Q.H.)

**Keywords:** marine sensor network, graph partitioning, multi-objective partition, genetic algorithm, incremental optimization strategy

## Abstract

Existing marine sensor networks acquire data from sea areas that are geographically divided, and store the data independently in their affiliated sea area data centers. In the case of marine events across multiple sea areas, the current network structure needs to retrieve data from multiple data centers, and thus severely affects real-time decision making. In this study, in order to provide a fast data retrieval service for a marine sensor network, we use all the marine sensors as the vertices, establish the edge based on marine events, and abstract the marine sensor network as a graph. Then, we construct a multi-objective balanced partition method to partition the abstract graph into multiple regions and store them in the cloud computing platform. This method effectively increases the correlation of the sensors and decreases the retrieval cost. On this basis, an incremental optimization strategy is designed to dynamically optimize existing partitions when new sensors are added into the network. Experimental results show that the proposed method can achieve the optimal layout for distributed storage in the process of disaster data retrieval in the China Sea area, and effectively optimize the result of partitions when new buoys are deployed, which eventually will provide efficient data access service for marine events.

## 1. Introduction

Marine sensor networks have become an important research field with the increasing global emphasis on marine rights and the marine economy. Marine sensor networks consist of various types of data acquisition equipment, such as buoys, subsurface buoys and voluntary observing ships. For example, in the famous Argo program [1], 12,081 buoys have been deployed in the ocean around the world since 1999, producing a huge amount of data stored by its affiliated data centers. These data provide the basic support for marine scientific research. Nevertheless, when dealing with marine events in multiple sea areas, this kind of storage method takes a long time to retrieve the data and affects real-time decision making, resulting in delays in emergency disaster prevention and rescue. It may even cause significant casualties and property loss.

Typhoons are typical marine events, the historical typhoon data since 1949 show that the main typhoons of the Chinese coastal areas land from the southern sea area of Taiwan and travel across several sea areas, including the East China Sea and the Philippine Sea [2]. When data are retrieved, common storage methods require access to multiple data centers, resulting in frequent data transmission and increasing communication cost. It also spawns some data processing problems such as scattered data, long retrieval time and low joining efficiency. Therefore, we need an optimal storage strategy that can support fast retrieval. A basic idea is to store data based on the relevance of ocean events. On the other hand, the highly correlated data is stored in the same node, reducing the cost of data retrieval. Nevertheless, the main difficulties of this data storage idea include two aspects: (1) data for different events have different degrees of correlation, and thus different data layouts can be drawn; (2) when new devices are deployed in the marine sensor network, the existing partition system needs to be updated.

In this paper, we first abstract the marine sensor network composed of all Argo buoys in the Chinese Sea area into Argo-buoys graph (AB-Graph), and use it as the main experimental object. Exploring the correlation of the buoys during previous disaster events is calculated to represent the edge weight of the AB-graph, we use the typhoon data of China’s coastal areas from 2000 to 2017. Then, we construct a multi-objective balanced partition method for AB-Graph, which divides the AB-Graph into multiple regions for efficient data storage and retrieval. AB-Graph partition principles are: maximizing the correlation of the buoy in the regions, minimizing the correlation of inter-regions, minimizing the communication time of inter-region and balancing the size of regions. Those principles aim to achieve an optimal layout for marine sensor network. This part of work has been published in the 2017 IEEE International Conference on Networking, Sensing and Control [3]. Furthermore, all kinds of new observation equipment are often deployed in the ocean. In order to deal with this situation effectively, this paper proposes an incremental optimization strategy, which can dynamically optimize the existing partition. Although our experiments are based on the Argo-buoy sensor network, the proposed method can be extended to a comprehensive marine sensor network with various types of sensors. For events across a number of sea areas, the method not only provides effective data retrieval for the relevant data calculation and analysis, but also continuously improves the quality of partition. It will help marine activities formulate decisions for disaster prevention quickly and enhance the safeguarding ability.

In this paper, Section 2 reviews and compares related work in graph partitioning and dynamic processing methods. Section 3 defines the AB-Graph and multi-objectives problems, and proposes a multi-objective balanced partition method based on NSGA-II. Section 4 introduces an incremental optimization strategy for post-partitioned AB-Graph. Section 5 presents four aspects of our experimental results. Finally, the paper is concluded in Section 6.

## 2. Related Work

With the rapid development of various marine sensing equipment, marine sensor networks can collect global ocean temperature, salinity, humidity and other information, which is widely used in marine event analysis, marine phenomena detection and other scientific research. Some researchers have built large-scale ocean data information systems to manage sensor data from storage equipment, storage environment, and data transmission [4,5,6,7], but they rarely consider the data storage methods so these methods take a long time for data retrieval. Novell and Palazov et al. [8,9] applied WMS service to provide near real-time retrieval to historical data for single-point access. However, they didn’t consider the relationship of sensors in the data management. Hedde et al. [10,11] computed the space-time correlation of sensor to manage complex wireless sensor networks. Meyerhenke et al. [12] used the graph partitioning method to divide the graph and then store it, effectively shortening the retrieval time. Taking the sensor correlation into consideration, Graph-based data storage strategies could enhance the efficiency of data retrieval. Therefore, this paper adopts the graph partitioning technology to process the marine sensor network data.

Graph partitioning can be used to cluster complex edge relations according to the correlation of vertices. In some data layout studies, a good graph partitioning algorithm can provide the basis for this. Common single-objective partitioning methods have used clustering algorithms and multi-level partitioning methods [12,13,14,15], but these methods might not give the best results [12,16]. Therefore, Damico et al. [17] proposed a single-objective simulated annealing algorithm (SA) for the police management problem in Buffalo (NY, USA). The method can help the police find the best patrol route and get high-quality solutions in the original graph. Rahimian et al. [16] firstly proposed a distributed graph partition algorithm, Ja-be-Ja1, which achieved better partition quality compared to METIS [18]. Later, some researchers used the vertex-cut partitioning of the large graphs by the parallel and distributed algorithm to optimize Ja-be-Ja1, named Ja-be-Ja-vc, which improved operation efficiency and performance in graph partitioning [19,20]. Compared with the hash partition, a graph partitioning method divided the graph using balanced label propagation and vertex migration strategies [21], which significantly reduced the execution time by more than 50% [22]. Nevertheless, the aforementioned single-objective partitioning methods had difficulty in meeting some practical needs in the production environment.

Multi-objective graph partitioning (MOGP) has been widely used in various fields, taking into account the impact of multiple factors on the graph and improving some objectives without reducing other objectives. MOGP methods are implemented using various multi-objective heuristic algorithms (MHA) [23,24]. Schloegel et al. [25] proposed a new formulation of the multi-objective graph partitioning problem, and designed an algorithm to compute partitioning with this formulation. This algorithm can handle similar and dissimilar objectives, finding a good tradeoff result, but it is highly sensitive to predetermined preference factors when forming integrated weights. In order to produce the solution in which both the cut and the maximum subdomain degree are simultaneously minimized, Selvakkumaran [26] proposed a family of multi-objective hypergraph partitioning algorithms, the maximum subdomain degree is reduced by up to 36% when compared with hMETIS [27]. Galvão et al. [28] proposed a multiplicatively-weighted Voronoi diagram (WVD) for treating the parcel delivery, and the experiment showed better results in both balanced time/capacity utilization. Jia et al. [29] combined Laplacian spectrum and self-organising map to propose a multi-objective power network partitioning approach, which obtained minimal intra-area real power imbalance with a healthy voltage profile. However the above methods have difficulty in performing fine-tuned control of tradeoffs among the objectives.

Some researchers have used multi-objective genetic algorithms to divide the graphs to find the optimal results [30,31,32]. They suggested that the graph partitioning should maximize the relation in the inner-subgraph and minimize the relation of the subgraphs. In order to improve the existing health care system in Parana (Brazil), the non-dominant sorting genetic algorithm (NSGA-II) has been used to distribute the medical institutions, and the experiment has proved that it has a good partitioning effect in the medical field [33]. NSGA-II [33] can design different operators for different fields, which has high scalability and it is easy to obtain high-quality solutions, but these static methods often take a lot of time to recalculate when the vertices or edges of the graph happen to change. Traditional dynamic processing methods generally use vertex migration strategies, such as the dynamic Mizan algorithm [34] mainly used to load balancing, and the xDGP algorithm [35] used to reduce the number of edge cuts. Similar strategies include the CANDS algorithm [36] and X-pregel algorithm for traffic networks [37]. Vaquero et al. [38] proposed an iterative vertex migration algorithm that only requires local information to reduce the execution time [22], which is suitable for scenarios where a large number of graph data changes exists. These mature multi-objective partition methods are widely used in the transportation, medical care, power grids, and other fields [19,32,39], but none of them has been used in data management for marine sensing networks. Therefore, this paper first proposes a multi-objective balanced partition method based on NSGA-II for marine sensor networks, which can maximize the correlation of sensors in the region, minimize the correlation of regions, minimize the communication time of inter-region, and balance the size of regions. This static method provides a basic storage method for sensing data. Then, we construct an incremental optimization strategy-based vertex migration to continuously optimize the results. Compared with other state-of-the-art graph and hypergraph partitioning methods such as MHP and WVD, our proposed method can easily obtain high-quality feasible solutions in large-size real-world situations and has a faster convergence speed. Typically, it can easily take fine-tuned controls among multiple objectives and get different priorities of the solution by user tendency. Facing complex environmental changes in marine sensor networks, the method can quickly give new partitioning results without significant degradation. It will provide fast and efficient data access service for time-changing ocean events.

## 3. Multi-Objective Balanced Partition Method for AB-Graph Based on NSGA-II

**Figure 1:**
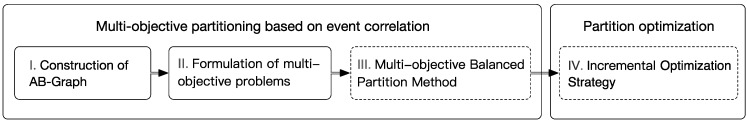
Workflow of the multi-objective partition method of marine sensor network based on degree of event correlation.

The workflow of the proposed method includes four parts (Figure 1). We describe the first three parts of workflow in this section. In Section 3.1, we define AB-Graph based on marine sensor network, and the multi-objective problems of marine sensor network are formulated. In Section 3.2, Section 3.3, Section 3.4 and Section 3.5, we describle a multi-objective partition method based on NSGA-II for AB-Graph. The last part of the workflow will be described in Section 4.

### 3.1. Construction of AB-Graph and Formulation of Multi-Objective Problems

#### 3.1.1. Construction of AB-Graph

In this paper, the marine sensor network composed of all Argo buoys in China Sea area is abstracted as an Argo-buoys graph (AB-Graph). AB-Graph = (V, E) is an undirected graph, where ${\;V\;=\;\{v}_{i};\;i\;=\;1,2,\ldots ,/V/\}$ is a set of |V| Argo vertices (buoys) in AB-Graph, and ${\;E\;=\;\{e}_{\mathrm{ij}};\;i,j\;=\;1,2,\ldots ,/V/;\;i\;\neq {\;j;\;e}_{\mathrm{ij}}{\;=\;e}_{\mathrm{ji}}\}$ is a set of |E| edges with $e_{\mathrm{ij}}$ as the edge between buoys $v_{i}$ and $v_{j}$. For more detailed information please refer to [3]. In the AB-Graph balanced partition problem, we design three objective functions and six constraints to divide the |V| Argo buoys of the Chinese sea area into MN regions ${\{M}_{1}{,\;M}_{2}{,\ldots ,\;M}_{\mathrm{MN}}\}$ and get the best way to store them. Table 1 shows the general notations used in this paper.

MN regions ${\{M}_{1}{,\;M}_{2}{,\ldots ,\;M}_{\mathrm{MN}}\}$ should be non-empty and non-intersecting, where $M_{k}{=\{v}_{1}^{\left(k\right)}{,\;v}_{2}^{\left(k\right)},\ldots \}$ is the subset of vertices in the AB-Graph at the $k_{\mathrm{th}}$ region. Table 2 lists the six constraints that partitioning the AB-Graph should meet.

#### 3.1.2. Formulation of Multi-Objective Functions

We design three objective functions to partition the AB-Graph. The objectives 1 and 2make the buoy of each region in the AB-Graph more close, i.e., the buoy with high correlation can be divided into the same region. Objective c controls the communication time cost by minimizing the number of regions involved in marine events.

1. Minimizing correlation of the buoys in the regions

The edge weight of the AB-Graph represents the degree of correlation between the two buoys. The sum of the edge weights in each region represents the correlation of the buoys in the region. When the sum of the edge weights of all regions reaches the maximum, the relationship of buoys is the closest in the AB-Graph, as shown in Equation (1):

(1) $$f_{1}=\overset{\mathrm{MN}}{\underset{k=1}{\sum }}\overset{\left|V\right|}{\underset{i=1}{\sum }}\overset{\left|V\right|}{\underset{j=1}{\sum }}C_{\mathrm{ik}}C_{\mathrm{jk}}\omega_{\mathrm{ij}}$$

where $\omega_{\mathrm{ij}}$ represents the edge weight between $v_{i}$ and $v_{j}$, the correlation of buoys in this region is most closely related to the maximization of the weight of the region. $\sum_{i=1}^{\left|V\right|}\sum_{j=1}^{\left|V\right|}C_{\mathrm{ik}}C_{\mathrm{jk}}\omega_{\mathrm{ij}}$ represents the sum of edge weights in the region $M_{k}$.

2. Minimizing the correlation of the buoys in inter-regions

Minimizing the correlation of inter-regions, i.e., the correlation within each region is maximized, and the sum of the weights of buoys within all regions is maximized. Therefore, this objective can also be achieved by Equation (1).

3. Minimizing the communication time of inter-regions

Communication cost of nodes is the same in the cloud platform, so the communication cost is mainly affected by the number of regions required for retrieval. We minimize the communication time of inter-regions by minimizing the average number of regions required for typhoon retrieval under this partition as shown in Equation (2):

(2) $$f_{2}=\frac{c}{\mathrm{Ty}}\overset{\mathrm{Ty}}{\underset{l=1}{\sum }}\overset{\mathrm{MN}}{\underset{k=1}{\sum }}d_{\mathrm{lk}}$$

where c indicates the communication time of the user accessing a single region in the cloud platform.

Through the above definition and description, we will construct a multi-objective balanced partition method based on NSGA-II. It consists mainly of four steps. First of all, we construct a unique representation and initialization method for AB-Graph, and then fill the mating pool with the binary tournament algorithm. Furthermore, we design a special selection, crossover and mutation operator for AB-Graph. Finally, we use the elite preservation mechanism of NSGA-II to preserve the optimal solution in each generation [33]. Each operator is described in Section 3.2, Section 3.3, Section 3.4 and Section 3.5.

### 3.2. Individual Representation and Initialization

It is difficult to obtain a feasible solution by using the traditional method of random assignment when initializing the individual (solution). In this paper, we use an array of |V| elements to represent the individual of the genetic algorithm, where the position of the elements in the array represents the serial number of the Argo buoy, and the value of the element represents the serial number of the region to which the buoy belongs. Then we design a greedy algorithm that is suitable for the marine sensor network, which is used to initialize the individuals that meet the constraints in Section 3.1.

The decomposition algorithm is shown in Algorithm 1. The computing complexity is O(1) for extracting MN vertex from the vertex set V and ${\mathrm{O;}(N}^{2})$ for traversing vertex and filling region. Thus, the complexity of Algorithm 1 is ${O(N}^{2})$.

**Algorithm 1**: Individual initialization algorithm**Input:** Argo buoys V in AB-Graph; The number of regions MN; Maximum size of region $M_{\max}$ **Output:** Individual of the population ${\mathrm{ind}}_{i}$ 1: The MN initial buoys are randomly selected and the value of the buoy in ${\mathrm{ind}}_{i}$ is set to k
 (k=1,2,…,MN); 2: while (All the buoys belong to a certain region) 3: if (The last round has successfully added the buoy to the region) 4:   for (Each region $M_{k}$) 5:    if (A vertex $v_{j}$, not belonging to any region, is connected to a vertex $v_{i}$ in $M_{k}$ and ${|M}_{k}{|<M}_{\max}$ )) 6:       Set the value of $v_{j}$ in the individual ${\mathrm{ind}}_{i}$ to be k; 7:      end if 8:    end for 9:  else 10:   for (Each region $M_{k}$) 11:     if ($v_{j}$ is not assigned and ${|M}_{k}{|<M}_{\max}$ ) 12:       Set the value of the buoy $v_{j}$ in the individual ${\mathrm{ind}}_{i}$ to be k; 13:    end if 14:  end for 15: end while 16: return ${\mathrm{ind}}_{i}$

### 3.3. Selection Operation

The selection operation algorithm is mainly based on the binary tournament algorithm. We judge the merits and demerits of the individual in the population through non-dominated sorting method [27]. The size of non-dominated level reflects the convergence of the solution, and the crowding distance reflects the diversity of the solution. Solution $S_{i}$ is better than solution $S_{j}$, if and only if Equation (3) is satisfied:

(3) $$\{\begin{array}{c} \delta_{S_{i}}{=\delta }_{S_{j}}{,\;\tau }_{S_{i}}{>\tau }_{S_{j}}\\ \delta_{S_{i}}{>\delta }_{S_{j}} \end{array}\;\mathrm{or}$$

where $\delta_{S_{i}}$ represents the non-dominance level of solution $S_{i}$, $\tau_{S_{i}}$ represents crowding distance of solution $S_{i}$. The selection operation algorithm takes two solutions (individuals) from the population and selects a solution with high convergence and diversity to fill the mating pool. This process is repeated until the mating pool reaches its predefined size, which is usually the same size as the population size. The time complexity of the fast non-dominated sorting is O(NlogN), and extended mating pool is O(N), so the time complexity of this algorithm is O(NlogN).

### 3.4. Cross Operation

**Figure 2:**
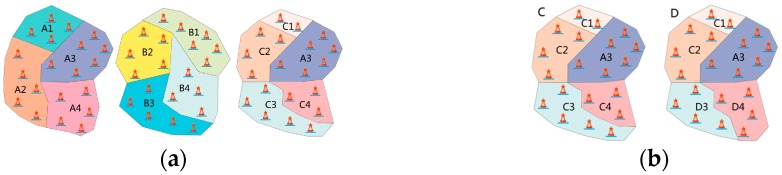
(**a**) Parents A and B generate a new solution C by crossover operation (**b**) Region C3 is changed by mutation operator.

When performing cross operation on individuals in the AB-Graph, we randomly select two individuals from the mating pool, select a certain region from a solution, copy and replace it into another solution, finally generate a new solution (descendant). Then we re-label the regions of the new solution, to prevent the overlapping within solution. As shown in Figure 2a, we assume that there are 20 buoys of the two solutions as parent A and parent B, which are labeled with four regions A1–A4, B1–B4, respectively. When the cross operation is executed, A3 in parent A is randomly selected to insert into parent B to generate a new solution. Since all the regions in B have A3 in common, the regions of new descendant are labeled as C1, A3, C2, C3, C4.

The decomposition algorithm is shown in Algorithm 2, where the time complexity of selecting two solutions randomly from the mating pool is O(1), region replacement is O(1), and re-labelling new solution is O(N). So the time complexity of this algorithm is O(N).

**Algorithm 2:** Cross operation algorithm for AB-Graph**Input:** Mating pool $P_{r}$ **Output:** Next-generation individual ${\mathrm{ind}}_{(r+1)i}$ 1: Two individuals ${\mathrm{ind}}_{\mathrm{ri}}$ and ${\mathrm{ind}}_{\mathrm{rj}}$ are randomly selected in $P_{r}$ 2: Randomly select a certain region in solution ${\mathrm{ind}}_{\mathrm{ri}}\left({\mathrm{ind}}_{\mathrm{rj}}\right)$, and replace the value of the position of the region in ${\mathrm{ind}}_{\mathrm{rj}}\left({\mathrm{ind}}_{\mathrm{ri}}\right)$ as the value in the ${\mathrm{ind}}_{\mathrm{ri}}\left({\mathrm{ind}}_{\mathrm{rj}}\right)$. The new solution is saved as a new individual ${\mathrm{ind}}_{(r+1)i}$ 3: The new individual ${\mathrm{ind}}_{(r+1)i}$ generated in 2 is re-labeled. It is judged whether the buoys in the same region are connected with each other, and if they are not connected, which will be labeled as a new region. 4: return ${\mathrm{ind}}_{(r+1)i}$

### 3.5. Mutation Operation

The principle of the mutation operator for AB-Graph’s individual is to randomly select some boundary buoys in a certain region and transfer each of them to another adjacent region, and then the original individual becomes a new individual without violating the constraints of Section 3.1. As shown in Figure 2b, the boundary buoy of C3 in individual *C* is transferred to its adjacent region C4, reducing the size of C3 but increasing the size of C4. Finally, all the regions that have changed are re-marked as D3, D4, resulting in a new individual *D*. The decomposition algorithm is shown in Algorithm 3 which has the time complexity of O(N).

**Algorithm 3:** Mutation operation algorithm for AB-Graph**Input:** Individual ${\mathrm{ind}}_{\mathrm{ri}}$ **Output:** Individual ${\mathrm{ind}}_{\mathrm{ri}}$ 1: Randomly select some border buoys ${\;V’=\{v’}_{i};\;i=1,2,\ldots ,/V’/\}$ belonging to one region $M_{i}(i=1,\;2\;,\ldots ,\;\mathrm{MN})$ in ${\mathrm{ind}}_{\mathrm{ri}}$; 2: for ($v_{j}$ in V’) 3: if ($N_{\mathrm{kj}}=1$ and $v_{j}\notin M_{i}$ and $v_{k}\in M_{i}$) 4:  The buoy $v_{j}$ is transferred to the region to which $v_{k}$ belongs; 5: end if 6: end for 7: Re-label the affected region; 8: return ${\mathrm{ind}}_{\mathrm{ri}}$

Finally, we apply the elite preservation strategy to NSGA-II [33], which can speed up the convergence rate of the algorithm and enhance the computing performance while retaining the outstanding solutions of each generation.

## 4. Incremental Optimization Strategy for the Post-Partitioned AB-Graph

Various types of sensors exist in the new deployment and decommissioning situation in the marine sensor network. After the buoy is deployed, its historical data needs to be retained, so AB-Graph is a typical incremental graph. This section only considers the increase of the buoys, and constructs the incremental optimization strategy for the post-partitioned AB-Graph by Section 4. This is divided into two steps: firstly, the expression factor of correlation between each buoy and each region is calculated by using the historical data of typhoon to indicate relationship of them; secondly, the incremental optimization algorithm for the post-partitioned AB-Graph is constructed to deal with the increasing change of buoys in the original partition of AB-Graph.

### 4.1. Expression Factor of the Correlation between Buoy and Region

In this paper, when a new buoy is added to AB-Graph, the expression factor of correlation is used to calculate the initial region, which is defined as follows:

**Definition 1.**  *The expression factor of correlation between the buoy and the region* $\varphi_{\mathrm{ik}}=(\sum_{t=1}^{\mathrm{Ty}}E_{\mathrm{it}})/(\sum_{l=1}^{\mathrm{Ty}}d_{\mathrm{lk}})$*, where* $E_{\mathrm{it}}$* indicates whether the buoy* $v_{i}$* is under the influence of the* $t_{\mathrm{th}}$* typhoon. If* $E_{\mathrm{it}}=1$*, the buoy* $v_{i}$* is affected by the* $t_{\mathrm{th}}$* typhoon, and* vice versa *when* $E_{\mathrm{it}}=0$*. If the value of* $\varphi_{\mathrm{ik}}$* is closer to 1, the correlation of the buoy* $v_{i}$* and the region* $M_{k}$* is more closely.*

### 4.2. Incremental Optimization Algorithm

AB-Graph’s incremental optimization algorithm consists of two parts. Firstly, according to Section 5.1, we calculate the correlation factor between the new buoy and the existing regions by definition 3, and then deploy it in the highest correlation region and fill the adjacent weight information. Secondly, we need to determine whether placing the new buoy into the AB-Graph will destroy the existing constraints of the partition. There are three cases: (1) when the new buoy is placed in a certain region in AB-Graph, the size of the region is larger than the pre-defined size, and there are other regions with spare size, then the existing partition is adjusted by vertex migration; (2) when the new buoy is placed in a certain region in AB-Graph, the size of the region is larger than the pre-defined size and the other regions are full, then the pre-defined size is expanded; (3) when the new buoy is placed in a certain region in AB-Graph, the size of the region is not larger than the pre-defined size, and it is directly placed in the region. Through the above three cases, our method satisfies the initial partitioning constraints and adapts to the influence of the change of the buoy on the existing partition, which improves the quality of partitioning result and limits the degree of degradation of the incremental optimization algorithm.

The decomposition algorithm is shown in Algorithm 4. Since the time complexity of the calculating expression factor is O(N), performing the vertex migration method is O(N), and Algorithm 3 has a time complexity of $O(\left|V_{r}\right|\times N)$.

**Algorithm 4**: Incremental optimization algorithm for the post-partitioned AB-Graph**Input:** Post-partitioned AB-Graph $G_{n}$; Set of new-added buoy $V_{r}$; Maximum size of region $M_{\max}$ **Output:** Optimized $G_{n+1}$ 1: for(Each buoy $v_{i}$ in $V_{r}$) 2:  for (Each region $M_{k}$) 3:    Calculate the value $\varphi_{\mathrm{ik}}$ when $v_{i}$ belongs $M_{k}$ by Definition 3; 4:  end for 5:  The buoy $v_{i}$ is placed in the region $M_{b}$ which has the maximum value $\varphi_{\mathrm{ib}}$, and expand its edge weight information; 6:  if(${|M}_{b}{|>M}_{\max}$ and other regions with spare size) 7:    Find the buoy $v_{c}$ with the smallest $\varphi_{\mathrm{cb}}$; 8:    for (Each region $M_{k}$) 9:      if (${|M}_{k}{|<M}_{\max}$) 10:        Calculate $\varphi_{\mathrm{ck}}$ by Definition 1; 11:      end if 12:    end for 13:    Place $v_{c}$ into the region with the maximum $\varphi_{\mathrm{ck}}$; 14:  else if (${|M}_{b}{|>M}_{\max}$ and other regions are full) 15:    Expand the value of $M_{\max}$; 16:  else if ((${|M}_{b}{|<M}_{\max}$) 17:    Maintain the existing partitions. 18:  end if 19: end for

## 5. Experiment

In order to evaluate our proposed method, this section first introduces the data and environment used in the experiment. Then, the changes of multi-objective balanced partition method are tested with varying population size, number of regions, quality of partitioning and running time. Thirdly, the performance of the multi-objective balanced partition method is compared with the traditional genetic algorithms and NSGA, and we analyze the effect of proposed method in different size of event data. Then, we compare the effects of incremental optimization strategies and other strategies. Finally, the performance of the method is evaluated.

### 5.1. Experimental Data and Environment

410 typhoon datasets were selected from the Typhoon Network [2] in 2000–2017. The 1.5 GB buoy data from China’s coastal is obtained from the Argo Real-Time Data Center [1]. Table 3 illustrates some examples of the typhoon data attributes.

Our experiments run on the cloud computing platform Spark 2.1.1. We build eight nodes as the storage sites for the partitioned regions. The communication between the nodes is through 10 Gigabit Ethernet.

### 5.2. The Partition Quality and Running Time by Different Population Size and Number of Regions

We build an AB-Graph with 53 vertices, 233 edges, and the sum of edge weights is 507. First, we test the number of iterations and find that when the number of iterations reaches 100, all populations have converged, so the maximum number of iterations in this experiment is no more than 100. Then according to the statistical analysis, we set the population size to 20, 50, 100, and the number of partitioning regions sets to 4–8. The degree of correlation of the buoys in the region $f_{1}$ is determined by the ratio θ:

(4) $${\;\theta =|E}_{S}|/|E|$$

In Equation (4),  |E| is the total edge weights of the AB graph. and $E_{S}$ is the sum of the preserving edge weights in all regions. That the larger value of θ is and the smaller the number of edges to be partitioned, indicates the higher the quality of the solution, and vice versa. It can be seen from Table 4 that with the increase of the population size, the overall quality of the partition is improved, indicating that the algorithm can more easily get high-quality solutions when the population size is larger. If the population size is 100, the number of regions is 4, the highest θ is 73.17%, which is 2.17% higher than the lowest, but its running time reached 24,479.152 ms, which is 6.01 and 2.8 times the running time when the population size is 20 and 50, respectively. This is because that when the population size increases, the algorithm needs to spend more time on initializing the individuals and filling the mating pool. In addition, according to Table 4, the running time of the algorithm decreases with the increase of the number of regions, which shows that the algorithm is more easily to converge and find the optimal solution as the number of regions increases.

### 5.3. Performance of the Proposed Method

In the experiment, we fix the population size as 100, divide the AB-Graph into four regions, and select all typhoon data from 2010 to 2017. The performance of the multi-objective balanced partition method is compared from two aspects. First, the proposed method compares with the traditional single-objective algorithm (SOGA), it can be seen from Table 5 that SOGA has obtained the highest quality solution 76.13% in θ, indicating that only 23.87% edge weights are partitioned, and the buoys of AB-Graph are closely related in the region, but its communication time across the region $f_{2}$ reached 153.34 ms. On the contrary, SOGA in $f_{2}$ can still get the optimal solution 121.21 ms, but its θ values is low, not satisfying our experimental expectations. Then, compared with the non-dominated sorting genetic algorithm (NSGA), the solution obtained by NSGA-II in this paper is 11.64% higher than NSGA solution in θ, and 4.14% in $f_{2}$. It is shown that the method proposed in this paper reduces the communication time across the region $f_{2}$ and significantly improves the correlation of buoys in the AB-Graph, meeting the expected original objectives of the experiment.

**Figure 3:**
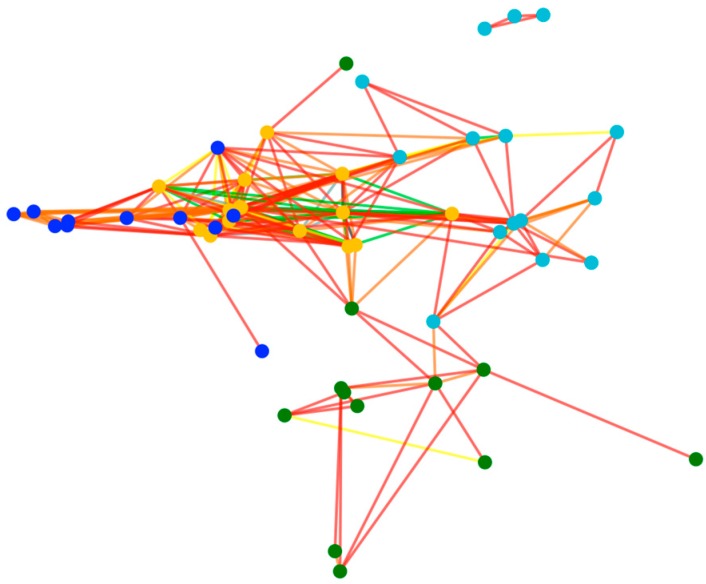
The results of the optimal solution.

The result of the optimal solution obtained in this paper is shown in Figure 3. All dots in the figure indicate 53 buoys in AB-Graph, and four colors of the vertices represent different regions, and the different colors of lines reflect the size of the edge weight.

We conduct further experiments to study the impact of data size on the performance of the proposed algorithm. We select 140 typhoon data from 2010 to 2015 as the basic event data and increase five typhoon data every time until 2017, and test the changes in the quality of the division. The experimental results are shown in Table 6.

It can be seen from Table 6 that the θ is slightly changed with the gradually increasing typhoons. Overall, with a large amount of event data, the method more easily obtains a high quality partition result, but the $f_{2}$ obviously decreases with the increase of typhoons, this is mainly due to the fact that the less the event data amount is, the simpler the structure of the AB-Graph is, and the shorter the communication time across the region $f_{2}$ is.

### 5.4. Effect Analysis of Incremental Optimization Strategy of Post-Partitioned AB-Graph

The experiment sets the population size to 100, the number of regions is set to 4, and selects all typhoon data from 2014 to 2016. Under this condition, the optimal solution is obtained by the multi-objective balanced partition method in Section 3, where the θ is 69.49% and the $f_{2}$ is 125.91 ms. On this basis, we compare incremental optimization strategy with a hash partitioning method and repartition method in three aspects: θ, $f_{2}$ and running time, analyzing the effect of incremental optimization strategy.

**Figure 4:**
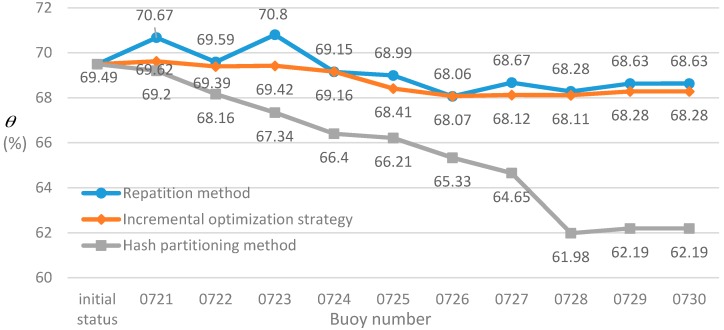
The change of the three methods on the θ when the buoy number increases.

First of all, we examine the effect of three processing methods in θ after new buoys are added to the AB-Graph, as shown in Figure 4. Currently, the AB-Graph has a total of 53 vertices. In order to limit the influence of the number of new added buoys on original graph, 20% of the total buoys are randomly generated in the Chinese sea area.

It can be seen from Figure 4 that the hash partitioning method is less concerned with the correlation of the existing AB-Graph vertices when the number of buoys increases, θ has a serious degradation. In the “0728” buoy placed, it reaches a minimum value of 61.98%, which is 7.51% lower than the initial state. The incremental optimization strategy proposed in this paper increases with the buoys which finds that θ is fluctuating around 68.68% and the trend is stable. When the “0721”, “0723”, “0727” and “0729” buoys are added to the graph, the θ starts to rise, indicating that the incremental optimization strategy can optimize the existing partition of the graph when adding new devices. The validity of the method is demonstrated. Compared with the repartition method, when the “0721”, “0723”, “0727”, “0729” buoys added to the graph a rebound is also produced. When the buoy number “0726” is placed into the AB-Graph, θ is only 0.01% difference, indicating that the incremental optimization strategy is similar to the repartition method on θ, and the result of incremental optimization strategy is reliable. Overall, the repartition method is only slightly higher than the incremental optimization strategy on the θ, which indicates that the incremental optimization strategy can be applied to accommodate the situation of changing buoy deployment..

**Figure 5:**
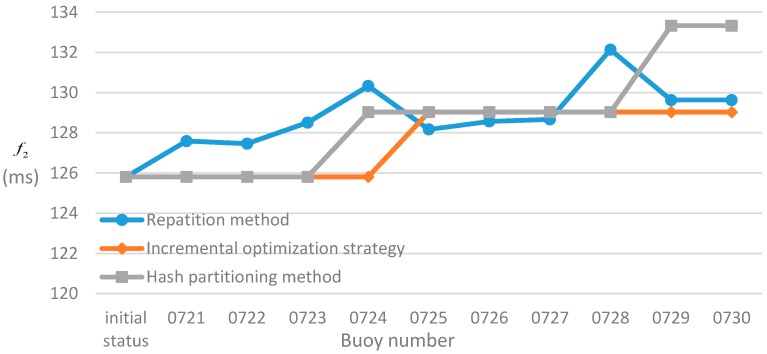
The change of the three methods on the $f_{2}$ when the buoy number increases.

Secondly, the three methods are compared with regards to the $f_{2}$ (see Figure 5). It can be seen from Figure 5 when the “0724” and “0729” buoys are placed into the graph, those $f_{2}$ of the hash partitioning method increase to 129.03 ms and 133.33 ms, and those $f_{2}$ growth are obviously comparable with the incremental optimization strategy. For the repartition method, those $f_{2}$ of the repartition method produce a significant fluctuation as the buoys are added into the graph. This indicates that the repartition method has higher instability and frequent vertex migration, which increases the communication cost of the production environment. Compared with the other two methods, the incremental optimization strategy is stable and can improve the partition quality of the AB-Graph step by step, and effectively control the number of vertex migration.

Finally, given that the population size is 100 and AB-Graph is divided into four regions, when we add a new buoy to AB-Graph, the average running time of the incremental optimization strategy is 11.40 ms, and the running time of the repartition method is 21,314.70 ms due to the fact that needs to re-calculate all the buoys and regions. It may be considered that when a large number of buoys (e.g., 1900 buoys) are placed at the same time, the repartition method will take a similar running time with the incremental optimization strategy but get higher partition quality. However, it should be noted that the repartition method may cause large-scale vertex migration cost.

### 5.5. Verification of the Partitioning Effect

We set different sizes of event data to test the partitioning effect, the experimental environment is similar as in Section 5.3. We count the ratio θ of buoys stored in the same region when typhoons are retrieved to reflect the partitioning effect, the detailed results are shown in Table 7.

The value of θ always fluctuates around 59.71%, when the number of typhoons is 140 and 160, 145 and 150 have the same partitioning quality, indicating that the proposed method has high stability. The proposed method can provide a more stable optimal result for different sizes of marine events, and provide reliable support for data layout.

The best partition of the AB-Graph obtained in Section 5.3 is shown in Table 8. The sizes of the four regions are similar, indicating that the result of proposed method is balanced. In order to verify the partitioning effect, we choose 170 typhoon data for statistical analysis from 2010 to 2017. Experiments show that when typhoons are retrieved, all buoy data associated with 59.15% and 26.76% of the typhoons are stored in the same region and two regions, respectively, the others need to access three or more regions.

## 6. Conclusions

In this paper, firstly, we analyzed the multi-objective partitioning problem of marine sensor networks. Secondly, the Chinese Argo buoys are abstracted into an AB-Graph as the main experimental object. Thirdly, we constructed the various operators for AB-Graph based on NSGA-II and partition the AB-Graph. Finally, an incremental optimization strategy is proposed to ensure that the existing partitioning results are optimized at a low cost when the number of devices in the AB-Graph changes.

In the verification of the multi-objective balanced partition method, we showed that compared with the traditional genetic algorithms and NSGA, the proposed method significantly improves the correlation of the buoys in the region, and reduces the communication time of inter-regions. Then, in order to validate the partitioning effect, we assumed that 85.91% of the typhoons need to access only one or two regions during the retrieval and the size of each region is balanced, the results showed that the proposed method can provide fast and efficient data access services in marine events. In addition, when various types of observation equipment are deployed, the incremental optimization strategy, compared to the hash partitioning method and repartition method, quickly adjust the existing partition, getting high-quality results. The shortcoming of this study lies in the fact that the impact of buoy location changes on the partitioning and hypersensitivity of marine data are ignored. Future work will focus on dynamical buoy layouts, and data security of the cloud computing platform.

## Figures and Tables

**Table 1:** Description of related parameters.

Parameter	Description of Parameter
Ty	Number otyphoons
$C_{\mathrm{ik}}=1$	The buoy $v_{i}$ belongs to the k th region, i=1,2,…,/V/; k=1,2,…,MN
$C_{\mathrm{ik}}=0$	The buoy $v_{i}$. does not belongs to the k th region, i=1,2,…,/V/; k=1,2,…,MN
$d_{\mathrm{lk}}=1$	The l th typhoon is related to buoys in the region $M_{k}$, l=1,2,…,Ty; k=1,2,…,MN
$d_{\mathrm{lk}}=0$	The l th typhoon is not related to buoys in the region $M_{k}$, l=1,2,…,Ty; k=1,2,…,MN
$N_{\mathrm{kj}}=1$	Buoy $v_{i}$ and buoy $v_{j}$ are connected to each other. i=1,2,…,/V/
$N_{\mathrm{kj}}=0$	Buoy $v_{i}$ and buoy $v_{j}$ are not connected to each other. i=1,2,…,/V/

**Table 2:** Description of constraints.

Description of Constraint	Constraint
Number of regions	${\mathrm{MN}}_{\min}\leq \mathrm{MN}\leq {\mathrm{MN}}_{\max}{;\;\mathrm{or}\;\mathrm{MN}=\mathrm{MN}}_{\mathrm{fix}}{;\;\mathrm{MN}}_{\min}>1;{\mathrm{MN}}_{\max}<|V|$
Size of region	$M_{\min}\leq {|M}_{k}|\leq M_{\max}{;\;M}_{\min}\geq {1;\;M}_{\max}\leq |V|;\;k=1,2,\ldots ,\mathrm{MN}$
Disjoint regions	$M_{1}\cap M_{n}=\emptyset \;\;\;\;l,n=1,2,\ldots ,\mathrm{MN};\;l\neq n$
Non-empty region	$M_{k}\neq \emptyset \;\;\;k=1,2,\ldots ,\mathrm{MN}$
Including all buoys	$M_{1}\cup {\ldots M}_{k}{\ldots M}_{\mathrm{MN}}=V$
Integrity of Buoy	$\sum_{k=1}^{\mathrm{MN}}C_{\mathrm{ik}}=1\;i=1,2,\ldots ,|V|$

**Table 3:** Samples of typhoon data.

No.	Start Date	End Date	Data 1		…	Data *k* (*k* Is Variable)	
200,001	5 May 2000 08:00	12 May 2000 20:00	Longitude, Latitude	135, 9.9	…	Longitude, Latitude	149.5, 28.4
			Wind Speed (m/s)	15		Wind Speed (m/s)	15
			…	…		…	…
			Pressure (dbar)	1004		Pressure (dbar)	998
			Wind-force (Category)	7		Wind-force (Category)	7
…	…		…	…	…	…	…
201,703	2 July 2017 08:00	4 July 2017 17:00	Longitude, Latitude	126.8, 20.3	…	Longitude, Latitude	136.3, 34.2
			Wind Speed (m/s)	18		Wind Speed (m/s)	23
			…	…		…	…
			Pressure (dbar)	1000		Pressure (dbar)	992
			Wind-force (Category)	8		Wind-force (Category)	9

**Table 4:** The relationship between θ and running time for each population size.

The Population Size	The Number of Regions	θ (%)	Running Time (ms)
20	4	**71.00**	4067.528
	5	64.69	3744.439
	6	59.17	3117.520
	7	50.49	3093.2761
	8	47.14	2503.271
50	4	**71.99**	8731.366
	5	66.46	8486. 331
	6	61.53	8392.4750
	7	56.01	8212.1418
	8	50.88	7645.235
100	4	**73.17**	24,479.152
	5	66.66	23,549.383
	6	59.56	21,753.301
	7	57.00	20,001.864
	8	55.02	1655.209

**Table 5:** Comparison of NSGA-II with other genetic algorithm in AB-Graph partition problem.

Objective Function	θ (%)	$f_{2}$ (ms)
SOGA with the maximum value of *θ*	76.13	153.34
SOGA with the minimum value of *f*_2_	57.59	121.21
NSGA solution	61.93	150.55
NSGA-II Optimal solution	73.57	144.31
Improvement	**11.64%**	**4.14%**

**Table 6:** Performance of proposed method in different data size.

The Number of Typhoons	θ (%)	$f_{2}$ (ms)
140	70.19	107.89
145	72.62	110.19
150	72.85	112.72
155	72.14	114.34
160	72.59	125.21
165	72.16	143.15
170	73.57	144.31

**Table 7:** Partitioning effect of proposed method by different data sizes.

The Number of Typhoons	θ (%)
140	61.11
145	59.72
150	59.72
155	61.11
160	58.94
165	60.27
170	59.72

**Table 8:** Optimal results of layout for AB-Graph.

Region ID	Number of Buoys	Buoy ID
1	15	“0124”, “0129”, “0143”, “0156”, “0259”, “0294”, “0146”, “0241”, “0086”, “0228”, “0158”, “0331”, “0332”, “0333”, “0338”
2	15	“0094”, “0144”, “0222”, “0233”, “0286”, “0149”, “0160”, “0290”, “0229”, “0225”, “0231”, “0234”, “0220”, “0224”, “0199”
3	12	“0187”, “0188”, “0221”, “0262”, “0264”, “0182”, “0151”, “0148”, “0184”, “0185”, “0072”, “0205”
4	11	“0196”, “0195”, “0194”, “0335”, “0181”, “0364”, “0365”, “0366”, “0367”, “0368”, “0359”
